# Effectiveness of the monitoring program for ensuring the quality of water treated for dialysis in the state of São Paulo

**DOI:** 10.1590/2175-8239-JBN-2018-0026

**Published:** 2018-08-02

**Authors:** Adriana Aparecida Buzzo Almodovar, Márcia Liane Buzzo, Fernando Pontes de Lima e Silva, Ellen Gameiro Hilinski, Adriana Bugno

**Affiliations:** 1 Instituto Adolfo Lutz, Núcleo de Ensaios Biológicos e de Segurança, São Paulo, SP, Brasil.; 2 Instituto Adolfo Lutz, Núcleo de Contaminantes Inorgânicos, São Paulo, SP, Brasil.; 3 Instituto Adolfo Lutz, Centro de Medicamentos, Cosméticos e Saneantes, São Paulo, SP, Brasil.

**Keywords:** Renal Dialysis, Water Quality Control, Water Monitoring, Program Development, Diálise Renal, Controle da Qualidade da Água, Monitoramento da Água, Desenvolvimento de Programas

## Abstract

**Introduction:**

Chronic kidney failure is a disease that affects the functions of the kidneys and can cause irreversible kidney failure over time. Among the main factors that cause this disease are hypertension and diabetes *mellitus*. The number of patients presenting this clinical condition has been increasing in Brazil, leading to an increase in renal replacement therapy, such as hemodialysis.

**Material and methods:**

In the state of São Paulo, a joint action between the Adolfo Lutz Institute, the Sanitary Surveillance Center, and the Sanitary Surveillance Groups have promoted the State Program for the Monitoring of Water Treated for Dialysis since 2007 to evaluate the chemical and microbiological quality of the water used in dialysis in compliance with the current legislation.

**Objective:**

This study aimed to evaluate the monitoring program developed between 2010 and 2016 as a tool for corrective action when unsatisfactory results are observed.

**Results:**

The level of satisfactory results during the period varied from 85.8 to 98.0%, indicating an increase in the adequacy of the dialysis services in producing water with adequate quality for patient health.

**Conclusion:**

The design adopted in the state monitoring program is highly effective based on new collections after the joint actions of the Sanitary Surveillance System and the State Dialysis Services.

## RESUMO

**Introdução:**

A Insuficiência Renal Crônica caracteriza-se como uma doença que afeta as funções dos rins, podendo causar a falência irreversível dos órgãos ao longo do tempo. Dentre os principais fatores que podem causar a doença, destacam-se a hipertensão arterial e o diabetes *mellitus*. O número de pacientes com esse quadro clínico - que precisam submeter-se a procedimentos de tratamentos renais substitutivos, como a hemodiálise - vem aumentando no país.

**Materiais e métodos:**

No estado de São Paulo, uma ação conjunta entre o Instituto Adolfo Lutz, o Centro de Vigilância Sanitária e Grupos de Vigilância Sanitária vem promovendo, desde 2007, o Programa Estadual de Monitoramento de Água Tratada para Diálise para avaliar a qualidade química e microbiológica da água utilizada em tratamentos dialíticos, em atendimento à legislação vigente.

**Objetivo:**

O presente estudo teve como objetivo avaliar o Programa de Monitoramento desenvolvido entre 2010 a 2016, como ferramenta para tomadas de ações corretivas quando resultados insatisfatórios foram observados.

**Resultados:**

O nível de resultados satisfatórios no período variou de 85,8% a 98,0%, indicando aumento de adequação dos Serviços de Diálise na produção de água com qualidade necessária à preservação da saúde dos pacientes.

**Conclusão:**

Os resultados indicaram que o delineamento adotado no programa estadual de monitoramento, com novas colheitas após tomadas de ações conjuntas entre os órgãos do Sistema de Vigilância Sanitária e as equipes dos Serviços de Diálise do estado, apresenta alta efetividade.

## INTRODUCTION

Chronic kidney failure (CKF) is defined as a progressive, slow, and irreversible deterioration of renal function concerning the elimination of toxic substances produced by the body, which accumulate in the blood. Its estimated prevalence of 8 to 16% has increased in the world population.^1^ Additionally, high mortality rate, morbidity rates, and financial costs are associated with the disease,^2^^-^ 6 which justifies the adoption of public health preventive measures.

In Brazil, chronic noncommunicable diseases (CNCDs), such as hypertension and diabetes *mellitus*, are the most important diseases for the development of CKF. These diseases are more prevalent in the age groups of 65 to 74 years and over 75 years, with prevalence rates of 52.7 and 55% for hypertension, and 19.9% and 19.6% for diabetes, respectively^7^. In the more advanced stage of CKF, patients may rely on renal replacement therapies (RRTs), including hemodialysis or peritoneal dialysis, or on transplants.^4^^,^ 7 ^-^ 10 In 2013, hemodialysis was the treatment of choice for 90% of chronic renal patients in Latin America, of whom 43% were from Brazil.^10^

Censuses performed by the Brazilian Society of Nephrology reveal a gradual increase in the number of chronic kidney patients in Brazil over the years. In 2016, the estimated number of patients undergoing dialysis treatment was 122,825. Of those, 113,122 underwent hemodialysis in 747 dialysis services in the country, 67% of whom were located in the southeast region.^7^ In 2015, the number of RRT services registered in the state of São Paulo was 190.^11^ Studies estimate a 28.4% increase in the number of patients undergoing weekly hemodialysis sessions in 2017.^5^

Hemodialysis is a widely used procedure for the treatment of renal deficiency in both its chronic and acute forms to normalize the electrolyte balance and remove toxic substances from the body through a dialysis solution composed mainly of water. Generally, the patient undergoes three weekly hemodialysis sessions and is exposed to approximately 120 liters of treated water at each session. Controlling the water quality used in the production of the dialysis solution is essential to avoid additional risks to the patient.^12^ Therefore, the water used in dialysis services must comply with the minimum requirements for the chemical and microbiological parameters defined in the current legislation (Resolution RDC No. 11/2014,^13^ which includes Good Operating Practice Requirements for Dialysis Services).

Considering the impact of water quality in dialysis on the patients' safety, the state São Paulo established a program to monitor the quality of treated water for dialysis in a joint action between the State Sanitary Surveillance Center, the Adolfo Lutz Institute, Sanitary Surveillance Groups of state, and the municipalities. The program was conducted continuously in all active dialysis services of the state of São Paulo.^14^

The objective of this study was to present the development of the Program for Monitoring Treated Water for Dialysis for the systematic evaluation of the standards of water quality treated in dialysis services in the state of São Paulo between 2010 and 2016. The analysis was based on the parameters recommended in the current legislation and the orientation of health actions to preserve the safety of patients undergoing dialysis treatments.

## MATERIAL AND METHODS

### SAMPLES

The samples were collected by the Sanitary Surveillance Groups at the state and municipalities from the dialysis services of the state of São Paulo.

The procedures for collecting, preserving, packaging, and transporting samples defined in the Manual for Water Analysis of the Adolfo Lutz Institute^15^ were used. The procedures were based on the recommendations of the American Public Health Association^16^ and were used to standardize the procedures adopted by the collecting teams and ensure the reliability of the analytical results. In addition, the teams were periodically trained on the sampling procedures during the monitoring program.

The Adolfo Lutz Institute also provided the material for collection, prepared specifically for each test: sterile vials for microbiological tests, depyrogenated vials for bacterial endotoxins, decontaminated and preservative-free bottles for the chemical tests, and chemically decontaminated bottles containing appropriate preservatives for the analyses of metallic contaminants and mercury. All materials were made available to the Sanitary Surveillance Groups according to a previously defined schedule in isothermal boxes containing reusable ice.

### COLLECTION POINTS

The sampling points were defined by the legislation at the time of each round of the Program. Between 2010 and February 2014, samples were collected at the reuse point for all assays as defined in the Resolution RDC No. 154/2004.^17^ From 2014 onwards, the Resolution RDC No. 11/2014^13^ defined that samples had to be collected at the exit of the water treatment system for the chemical tests and the determination of metals and mercury and at the point of reuse for the microbiological evaluation and bacterial endotoxin assessment.

### ANALYTICAL METHODS

The laboratory work was conducted at the Adolfo Lutz Institute considering the parameters defined in the legislation as follows:

- Microbiological analysis: counting of heterotrophic bacteria (pour plate in R2A agar, incubation at 36°C for 96 h) and total coliforms (presence-absence method);
- Bacterial endotoxins: *Limulus* Amebocyte Lysate (LAL) - gel cloth method;
- Chemical analyses: nitrate (ultraviolet spectrophotometry), sulfate (turbidimetry), fluoride (potentiometry with selective electrode), conductivity, and pH;
- Determination of metals: aluminum, antimony, arsenic, barium, beryllium, cadmium, calcium, lead, copper, chromium, magnesium, potassium, silver, selenium, sodium, thallium, and zinc (inductively coupled argon plasma mass spectrometry);
- Determination of mercury (cold vapor atomic absorption spectrometry).

### OUTLINE OF THE PROGRAM

Between 2010 and 2014, the Monitoring Program consisted of an initial collection from all active dialysis services in the state of São Paulo and a second collection only from the services that presented an unsatisfactory parameter in the first collection. As of 2015, the Program performed up to three collections, in addition to the initial one, from clinics that presented an unsatisfactory parameter in the first round to evaluate the effectiveness of the corrective actions performed by the dialysis services to adapt their water treatment systems.

## RESULTS

Table 1 shows the number of dialysis services of the state of São Paulo that were evaluated between 2010 and 2016 and the frequency of new sample collections due to a previous inadequate parameter. During the study period, some active clinics were not evaluated due to changes in address, closure of activities not reported to the Sanitary Surveillance System, or a technical problem detected by the Surveillance Group teams.

**Table 1:** Dialysis services evaluated in the Monitoring Program between 2010 and 2016

Year of execution	Number of dialysis services					Total analyzed samples
	Active	Evaluated				
		First collection	Second collection	Third collection	Fourth collection	
2010	175	169	33	-	-	202
2011	179	174	29	-	-	203
2012	172	168	28	-	-	196
2013	182	151	18	-	-	169
2014	189	183	18	-	-	402
2015	184	184	33	13	01	448
2016	193	193	41	17	09	494

Table 2 presents data of quality of water in the dialysis services from surveillance programs in other states of Brazil.

**Table 2:** Monitoring of the quality of water treated used in dialysis treatment in different states of Brazil

Place of study	Year of execution	^1^ Unsatisfactory results (%)		Reference
São Paulo	2007	49.0		14
São Paulo	2008	38.7		12
	2009	28.7		
Rio de Janeiro	2008 to 2010	27.3		18
Distrito Federal	2009 to 2010	21.8		19
Bahia	2012	31.0		20
Rio Grande do Norte	2012 to 2013	100.0		21
**Place of study**	**Year of execution**	**^1^ Unsatisfactory results (%)**		Present study
		**First collection**	**Year**	
São Paulo	2010	20.1	5.3	
	2011	16.7	5.2	
	2012	17.3	6.6	
	2013	12.6	2.0	
	2014	20.2	14.2	
	2015	17.9	6.6	
	2016	27.5	9.9	

1 At least one parameter that does not comply with the current legislation.

**Figure 1:**
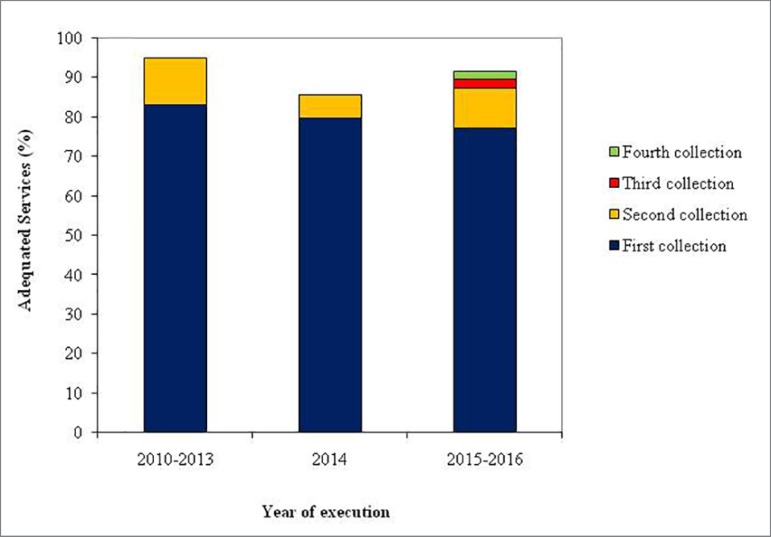
Frequency of services considered adequate for the quality of water treated used for dialysis evaluated by the State Monitoring Program.

Figure 1 shows the distribution of dialysis services of São Paulo that presented water treatment and distribution systems in accordance with the standards defined by the current legislation.

**Figure 2:**
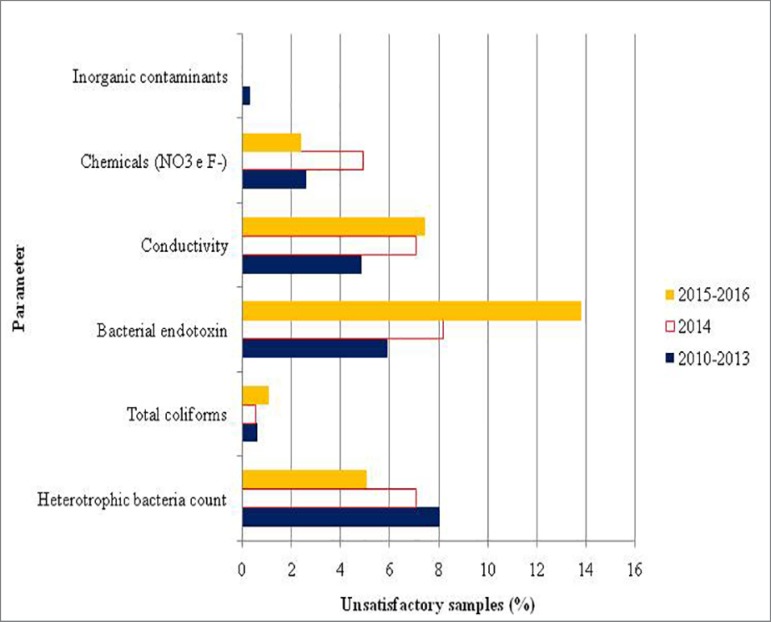
Frequency of unsatisfactory results determined at the initial sample collection as a function of the analyzed parameter

**Figure 3:**
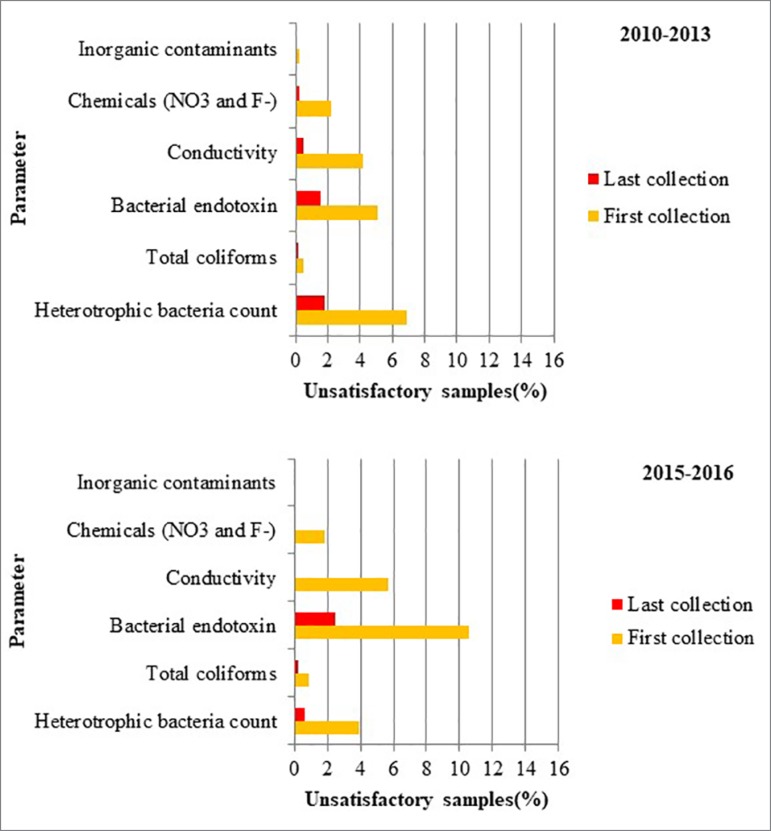
Comparison of between unsatisfactory results of the initial and last sample collection as a function of the analyzed analytical parameter.

Considering the results obtained in the first collection of treated water samples in the dialysis services of the state of São Paulo, Figure 2 presents the incidence of inadequate parameters according to the maximum limits allowed in the legislation and Figure 3 show a comparison between unsatisfactory results recorded in the first and last collections of the Program.

## DISCUSSION

The State Program for Monitoring the Quality of Water Treated for Dialysis has been conducted periodically to test and evaluate the public health risk of the of the water treated in the state's dialysis services and to ensure that the quality of the water complies with the standards established in the current legislation.

The study evaluated the results obtained between 2010 and 2016. However, the water quality parameters were different during the periods from 2010 to 2013 and from 2015 to 2016 due to the publication of Resolution RDC No. 11/2014,^13^ which established different limits in relation to the earlier legislation^17^ for microbiological parameters and bacterial endotoxins. Additionally, because there was a six-month period between March and September 2014 for the transition between the previous and current legislation, data from 2014 were excluded from the analysis.

Between 2010 and 2013, the percentage of dialysis services that presented satisfactory results in the first sample collection ranged from 80% in 2010 to 87% in 2013. During the following period, from 2015 to 2016, a decrease was observed in the number of clinics in compliance with the current water quality parameters to 77% on average (Figure 1).

Previous studies^12^^,^ 14 ^,^ 18 ^-^ 21 about the quality of water used in treatment of CKF patients in the country (Table 2) found higher frequencies of unsatisfactory results than the present study, even when the results of the first sample collection of the year were compared.

Because the first collection served as an initial snapshot of the water quality and distribution system in the dialysis service, each round of the Program presented variations in the incidence of parameters in disagreement with the maximum limits allowed in the legislation in force at the time (Figure 2). Bacterial endotoxins, conductivity, and chemical parameters had the highest incidences of unsatisfactory results at each round of the Program.

The worse results observed between 2015 and 2016 compared to the period from 2010 to 2013 is related to the more strict limits of the 2014 Resolution^13^ for microbiological parameters (from 200 CFU/mL to 100 CFU/mL) and bacterial endotoxins (from 2 EU/mL to 0.25 EU/mL) compared to the previous legislation. These limits required the dialysis services to adjust their water treatment and distribution systems to meet the new quality standards.

In addition to the restriction of the current legislation, the increase of unsatisfactory results also coincided with the water crisis in the state of São Paulo due to drought between 2014 and 2015.^22^ Water scarcity during this period affected the water levels, caused serious social problems, and affected industry, agriculture, and the operation of basic institutions, such as hospitals and schools.^22^^,^ 23 The sustained reduction of water reserves in the state for an extended period affected millions of people and altered the routines of health services and infrastructures.^24^ According to the document Natural Disasters and Health in Brazil,^25^ the low rainfall index affected the quantity and quality of the water consumed by the population through eutrophication and algal blooms from catchment sources, intrusion of salt water into groundwater supplies, and biological contamination and chemical accumulation in the soil.^24^^,^ 25 Moreover, redistribution of water from different regions to compensate for the drop in reservoirs, the mixing of water from various supply systems, the consumption of the dead volume behind dams, the intermittent supply of water, and the depressurization of the distribution network may have compromised water quality by making water distribution systems and alternative sources of supply more vulnerable to external contamination.^26^

The first collection of each annual round of the Program was intended to evaluate the adequacy of the water treatment and distribution systems. The surveillance was performed in accordance with the conceptual, technical, and operational delineation elaborated by the central levels of the Adolfo Lutz Institute and Sanitary Surveillance Center. The analytical results of the first collection were used as a tool for corrective actions when at least one parameter was in disagreement with the current quality standard. Based on the unsatisfactory report, the municipal or state Sanitary Surveillance Group responsible for the sample collection returned to the dialysis service with the clinical team to develop strategies and adjust the water treatment and distribution system. Additionally, the teams established effective protocols for the water supply with an appropriate quality standard for dialysis.

The data obtained in this study indicated that despite the increase in the incidence of unsatisfactory results during the period from 2015 to 2016 for the first sample of the Program, the sanitary actions and conducts taken to guarantee the quality and safety of dialysis treatment were effective. Reductions in the percentage of unsatisfactory samples were observed between the first and the last sample collections performed between 2010 and 2013 and between 2015 and 2016 for all evaluated parameters (Figure 3). The heterotrophic bacteria count was reduced by 74% between the first and last collections for the period from 2010 to 2013 and by 84% for the period from 2015 to 2016. The detection of bacterial endotoxins was reduced by 69% during the first period and by 77% during the second period. The conductivity and chemical parameters were reduced by 88% during the first period and by 100% during the second period. These data reinforce that the design adopted by the state monitoring program is highly effective, with new collections occurring after joint actions between the organs of the Sanitary Surveillance System and the teams of the State Dialysis Services.

## CONCLUSION

Considering that water quality may impacts the morbidity and mortality of CKF patients undergoing dialysis treatments, the results of this study demonstrate that the systematic monitoring of dialysis services state of São Paulo by the Sanitary Surveillance System allows continuous improvement of the water treatment and distribution systems used by these services. The results also reinforce the importance of keeping the Monitoring Program as a tool to support joint actions between the Health Surveillance System and Health Services, to increase the effectiveness of water treatment and distribution systems used in dialysis and minimize the risks associated with dialysis treatment.
